# The enhancer activity of long interspersed nuclear element derived microRNA 625 induced by NF-κB

**DOI:** 10.1038/s41598-021-82735-x

**Published:** 2021-02-04

**Authors:** Hee-Eun Lee, Sang-Je Park, Jae-Won Huh, Hiroo Imai, Heui-Soo Kim

**Affiliations:** 1 Department of Integrated Biological Science, Pusan National University, Busan, 46241 Republic of Korea; 2 Institute of Systems Biology, Pusan National University, Busan, 46241 Republic of Korea; 3 National Primate Research Center, Korea Research Institute of Bioscience and Biotechnology, Cheongju, 28116 Republic of Korea; 4 Department of Functional Genomics, KRIBB School of Bioscience, Korea University of Science and Technology (UST), Daejeon, 34113 Republic of Korea; 5 Department of Cellular and Molecular Biology, Primate Research Institute, Kyoto University, Inuyama, Aichi 484-8506 Japan; 6 Department of Biological Sciences, College of Natural Sciences, Pusan National University, Busan, 46241 Republic of Korea

**Keywords:** miRNAs, Transcriptional regulatory elements, Mobile elements

## Abstract

Transposable elements (TEs) are DNA sequences that cut or introduced into the genome, and they represent a massive portion of the human genome. TEs generate a considerable number of microRNAs (miRNAs) are derived from TEs (MDTEs). Numerous miRNAs are related to cancer, and hsa-miRNA-625 is a well-known oncomiR derived from long interspersed nuclear elements (LINEs). The relative expression of hsa-miRNA-625-5p differs in humans, chimpanzees, crab-eating monkeys, and mice, and four primers were designed against the 3′UTR of *GATAD2B* to analyze the different quantities of canonical binding sites and the location of miRNA binding sites. Luciferase assay was performed to score for the interaction between hsa-miRNA-625 and the 3′UTR of *GATAD2B*, while blocking NF-κB. In summary, the different numbers of canonical binding sites and the locations of miRNA binding sites affect gene expression, and NF-κB induces the enhancer activity of hsa-miRNA-625-5p by sharing the binding sites*.*

## Introduction

Transposable elements (TEs) are genetic elements discovered by Barbara McClintock that exhibit mobility and replicability within the genome^1,2^. TEs can be classified into two classes, class 1 retrotransposons and class 2 DNA transposons. Class 1 retrotransposons have the ability to ‘copy-and-paste’, and class 2 DNA transposons can ‘cut-and-paste’ into the genome^3^. Approximately 90% of the plant genome and half of the mammalian genome harbor TEs. The precise functions of TEs have not been identified yet, although numerous studies have shown that TEs are involved in epigenetics, evolution, and human diseases^3–8^. Retrotransposons can be divided into three subclasses, i.e., non-long terminal repeats, long terminal repeats (LTRs), and *Dictyostelium* repetitive sequences (DIRSs). The three subclasses of retrotransposons are divided into the following: *Alu*, long interspersed nuclear element 1 (LINE1 or L1), short interspersed nuclear element (SINE)-variable number of tandem repeat (VNTR)-*Alu* (SVA), and human endogenous retrovirus K (HERV-K) and are active TEs in recent human evolution^5,9,10^. LINEs comprise approximately 20% of humans and include a 5′ untranslated region (UTR), two open reading frames (ORFs), and a 3′UTR with a polyadenylic acid (poly A) tail^5,9,10^. The expression of LINEs has been identified in various cancers, including lung, colon, and breast cancer as well as esophageal adenocarcinoma^11–13^. According to numerous reports, TEs generate gene regulators such as microRNAs (miRNAs) and transcription factors (TFs) due to TE insertion in the host genome. miRNAs derived from TEs are called MDTEs^14,15^.

miRNA is a regulator of gene expression that is made up of 19 to 24 nucleotides of small non-coding RNA molecules^16–18^. The known fundamental role of miRNA is to repress gene expression by complementary binding to the 3′UTR of the target gene. On the other hand, several studies have reported that miRNAs can bind to the 5′UTR and coding sequence (CDS), and miRNAs can both inhibit and enhance gene expression^19–23^. Hsa-miRNA-625 is well known as a cancer-related miRNA, oncomiR^24–28^ and hsa-miRNA-625 is derived from LINEs. hsa-miRNA-625 downregulates the target genes related to several cancers, including gastric, thyroid, esophageal, breast, and liver cancers^25–28^.

Both TFs and miRNAs contribute to gene regulation, and they are the major elements that support evolutionary aspects^29,30^. TFs bind to the nearby DNA to act as activators or repressors to enhance or repress gene transcription^31^. Nuclear factor kappa-light-chain-enhancer activated B cells (NF-κB) is a TF that is involved in immunity, cell survival, and cytokine production. Several studies have revealed that NF-kb, human response-related gene family, and interleukins (ILs) are important enhancer/activators in human immunodeficiency virus (HIV)^32–34^. miRNA and NF-κB studies revealed the NF-κB-miRNA network, which provides a better understanding of the regulation and cooperation of inflammation and cancer in various cancer types^35–37^. In addition to inducing expression, miRNAs repress NF-κB by direct targeting^38^.

The purpose of this study was to investigate the effect of different quantities of canonical sites and how the location of miRNAs complementary to the target gene will affect gene expression. The relative expression of hsa-miRNA-625-5p and its target gene *GATAD2B* in humans, chimpanzees, monkeys, and mice was also compared for evolutionary profiling. The quantity of canonical sties and the location of hsa-miRNA-625-5p influences the expression of *GATAD2B* in all tested species. Furthermore, the TF shares the sequence with one of the hsa-miRNA-625-5p, and the TF provides the enhancer activity of hsa-miRNA-625-5p. In this study, the idea that MDTEs provide TF binding sites (TFBSs) and that they collaborate to increase the power of super-enhancer miRNA is supported.

## Results

### Bioinformatic analyses of hsa-miRNA-625-5p and *GATAD2B*

**Figure 1:**
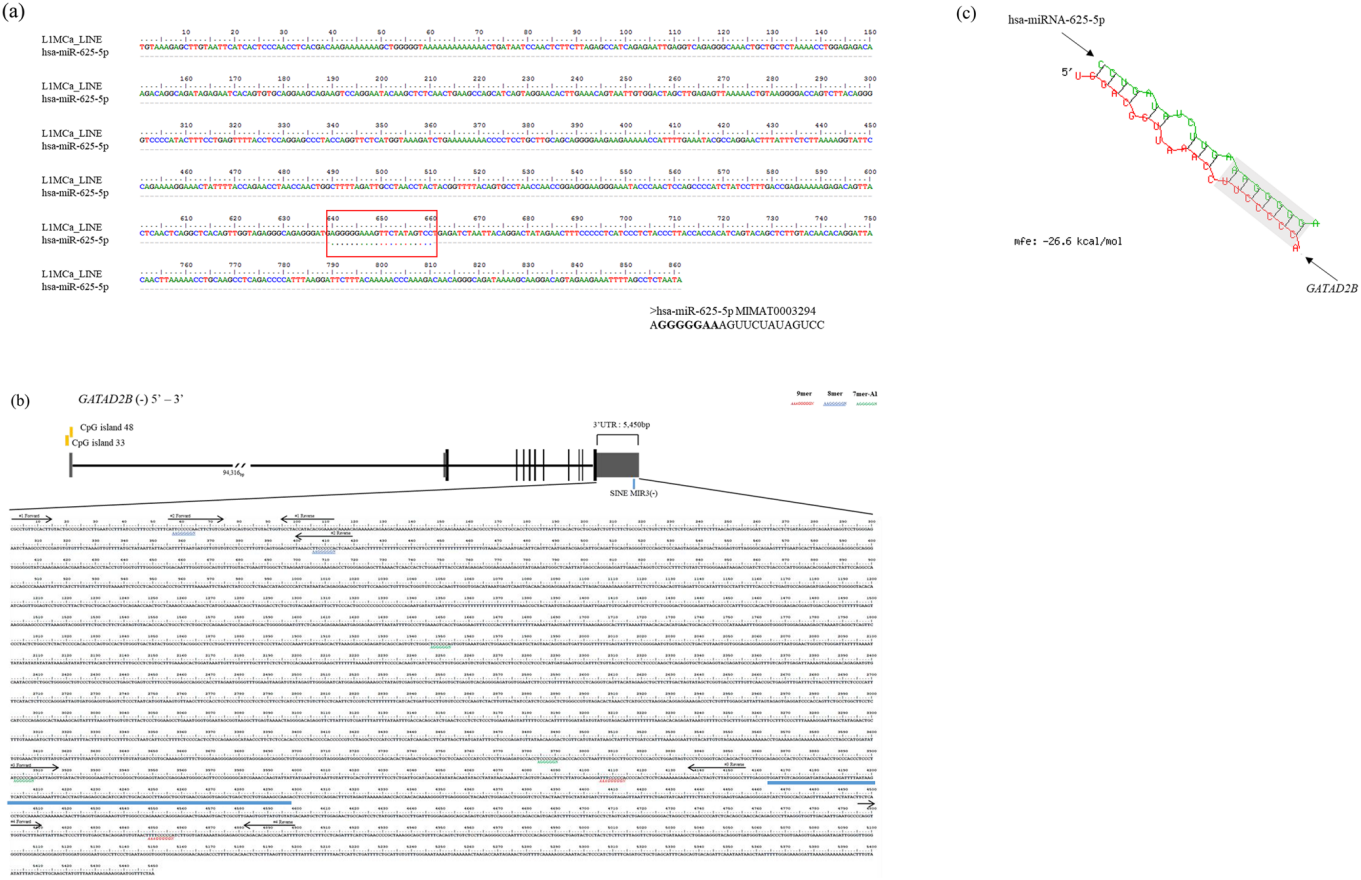
(**a**) The alignment result of LINE-L1MCa and hsa-miRNA-625-5p. The red box shows the conserved region of hsa-miRNA-625-5p and LINE-L1MCa. The hsa-miRNA-625-5p sequence is shown under the alignment, and the seed region is in bold. (**b**) Schematic structure of *GATAD2B* and the alignment result. The CDS regions are in black, and UTRs are grey. Two CpG islands are located in the 5′UTR of *GATAD2B*. In the 3′UTR of *GATAD2B*, there were seven binding sites of hsa-miRNA-625-5p. Additionally, one of the transposable elements, SINE MIR3, is located in the 3′UTR of *GATAD2B*. In the 3′UTR of *GATAD2B* sequences, 9mer hsa-miRNA-625-5p is indicated as italicized *AAAGGGGGN* in red, 8mer is indicated as underlined AAGGGGGN in blue, and 7mer-A1 is indicated as AGGGGGN in green. Additionally, four primers designed in the 3′UTR of *GATAD2B* are indicated in black arrows with each number and directions. (**c**) Bioinformatic analysis of hsa-miRNA-625-5p and *GATAD2B*. RNA hybrid of hsa-miRNA-625-5p and 3′UTR of *GATAD2B*. The seed region of hsa-miRNA-625-5p and complementary binding site of *GATAD2B* 3′UTR are in the grey box. The mfe value between hsa-miRNA-625-5p and *GATAD2B* 3′UTR is -26.6 kcal/mol.

The target gene list of hsa-miRNA-625-5p was downloaded from TargetScanHuman, and the candidates were selected based on the highest number and the length of the seed region that complementarily binds to the 3′UTR of the target gene (Table 1). The GATA zinc finger domain containing 2B (*GATAD2B*) gene had the highest number of 8mer hsa-miRNA-625-5p binding sites and the total binding sites in the 3′UTR (Fig. 1a). The schematic structure of *GATAD2B* shows that it is inserted into human genome in minus strand, and there two CpG islands near the 5′UTR (Fig. 1b). The 3′UTR of *GATAD2B* is 5450 bp long and contains two 9mer, two 8mer, and three 7mer-A1 of hsa-miRNA-625-5p complementary binding to 3′UTR sequences. Additionally, 3′UTR of *GATAD2B* includes one of transposable element called SINE MIR3 which is inserted into the human genome minus strand. To determine the differences between the quantity of canonical sites and the location of hsa-miRNA-625-5p binding sites, four primers were designed. Primer #1 contains only one 8mer hsa-miRNA-625-5p binding site in the front region of the 3′UTR, and primer #2 consists two 8mer of hsa-miRNA-625-5p binding sites in the front region of the 3′UTR. Primer #3 has one of the 9mer and 7mer-A1 hsa-miRNA-625-5p binding sites, and primer #4 includes one 9mer hsa-miRNA-625-5p binding site on the back side of the 3′UTR. Using the BiBiServ RNAhybrid, the structural interaction between hsa-miRNA-625-5p and the 3′UTR of *GATAD2B* was established (Fig. 1c). The minimum free energy (MFE) value between hsa-miRNA-625-5p and *GATAD2B* is − 26.6 kcal/mol. The seed region of hsa-miRNA-625-5p is well conserved when RNAhybrid is with the 3′UTR of *GATAD2B*.

**Table 1:** Target gene candidate list of hsa-miRNA-625-5p. *GATAD2B* gene in bold was selected as hsa-miRNA-625-5p target gene.

Gene name	Abbreviation	Location	Transcript ID	Total sites	8mer	7mer-m8	7mer-A1	6mer
Tetraspanin 11	*TSPAN11*	chr12:30,926,904 -30,996,602	ENST00000261177.9	6	3	1	1	0
**GATA zinc finger domain containing 2B**	***GATAD2B***	**chr1:153,804,907** **-153,922,975**	**ENST00000368655.4**	**7**	**4**	**0**	**3**	**0**
Sprouty-related, EVH1 domain containing 3	*SPRED3*	chr19:38,390,200 -38,399,883	ENST00000587013.1	4	3	0	1	3
Abl-interactor 2	*ABI2*	chr2:203,328,239 -203,432,173	ENST00000295851.5	6	4	1	1	1
Sperm Autoantigenic protein 17	*SPA17*	chr11:124,673,844 -124,694,791	ENST00000532692.1	4	3	0	1	1
B and T lymphocyte associated	*BTLA*	chr3:112,463,966 -112,499,561	ENST00000334529.5	4	3	0	1	0

Each column of table shows the name of the target gene, abbreviation of the gene name, the location in the human chromosome, transcript ID, total hsa-miRNA-625-5p binding sites in 3′UTR, total binding sites for 8mer, total binding sites for 7mer-m8, total binding sites for 7mer-A1 and total binding sites for 6mer.

### Analysis of the relative expression of hsa-miRNA-625-5p and *GATAD2B* in humans, male western chimpanzees, female western chimpanzees, female crab-eating monkeys, and male mice

**Figure 2:**
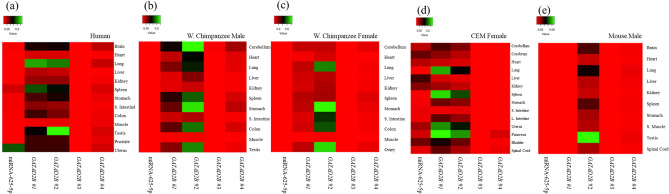
The relative expression analyses of hsa-miRNA-625-5p and *GATAD2B* by heatmap. (**a**) Heatmap of relative expression on hsa-miRNA-625-5p and 4 primers of *GATAD2B* in human. (**b**) Heatmap of relative expression on hsa-miRNA-625-5p and 4 primers of *GATAD2B* in male western chimpanzee. (**c**) Heatmap of relative expression on hsa-miRNA-625-5p and 4 primers of *GATAD2B* in female western chimpanzee. (**d**) Heatmap of relative expression on hsa-miRNA-625-5p and 4 primers of *GATAD2B* in female crab-eating monkey. Crab-eating monkey is shortened as CEM. (**e**) Heatmap of relative expression on hsa-miRNA-625-5p and 4 primers of *GATAD2B* in male mouse.

The relative expression of hsa-miRNA-625-5p and *GATAD2B* was analyzed using qPCR and then reanalyzed with Heatmapper (Fig. 2). The uterus had the highest relative expression of hsa-miRNA-625-5p in human samples (Fig. 2a), western chimpanzee males had the highest relative expression of hsa-miRNA-625-5p in the stomach and cerebellum (Fig. 2b), western chimpanzee females had the highest relative expression of hsa-miRNA-625-5p in small intestine (Fig. 2c), crab-eating monkey females had the highest relative expression of hsa-miRNA-625-5p in the liver (Fig. 2d), and mouse males had the highest relative expression of hsa-miRNA-625-5p in the heart (Fig. 2e). With respect to *GATAD2B* #1, humans (Fig. 2a) and western chimpanzee males (Fig. 2b) and females had the lowest relative expression in the muscle (Fig. 2c); crab-eating monkey females had the lowest relative expression in the small intestine (Fig. 2d); and male mice had the highest expression in the spleen and lowest expression in the brain and testis (Fig. 2e). However, with respect to *GATAD2B* #2, the results showed that the testis had the highest relative expression in humans. Western chimpanzee males had the highest *GATAD2B* #2 expression in the cerebellum, stomach, colon, and testis. Western chimpanzee females had the highest *GATAD2B* #2 expression in the lung, stomach, and ovary. The pancreas and spleen tissues had the highest relative expression in crab-eating monkeys. The testis of male mice showed the highest expression, and the lowest was observed both the kidney and heart (Fig. 2e). *GATAD2B* #3 showed the highest relative expression in the human prostate and uterus (Fig. 2a). The cerebellum in western chimpanzee males (Fig. 2b) and lungs in western chimpanzee females showed the highest relative expression of *GATAD2B* #3 (Fig. 2c). Similar to *GATAD2B* primer #1 and #2, the muscle showed the lowest expression in all three species. The crab-eating monkey had the highest relative expression of *GATAD2B* #3 in the womb and pancreas (Fig. 2d). Mice had the highest expression in the lung and lowest in the liver (Fig. 2e). Lastly, humans had the highest relative expression of *GATAD2B* #4 in the brain, lung, and uterus (Fig. 2a). The highest relative expression of *GATAD2B* #4 was observed in the cerebellum of western chimpanzee males (Fig. 2b) and the cerebellum, spleen, and stomach of western chimpanzee females (Fig. 2c). Female crab-eating monkeys had the highest relative expression of *GATAD2B* #4 in the pancreas (Fig. 2d). The relative expression of *GATAD2B* #4 in mice was not noticeably high or low in all 10 tissues (Fig. 2e). Again, western chimpanzee males and females showed the lowest relative expression of *GATAD2B* #4 in the muscle.

**Figure 3:**
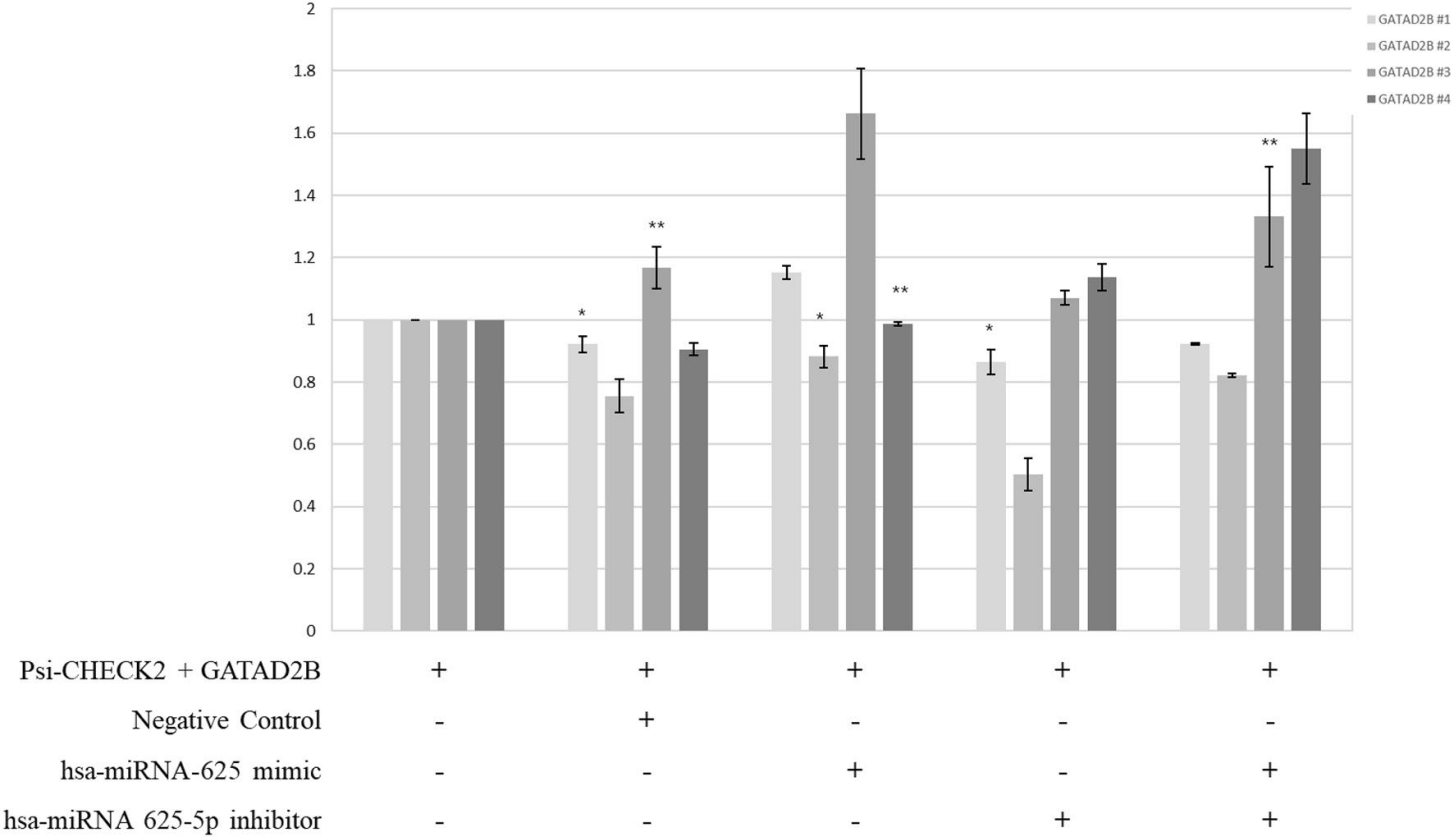
Luciferase analysis of hsa-miRNA-625-5p and *GATAD2B* in A549 cells*.* All the bars in the graphs were plotted as the mean ± SD. The student’s t-test was used to determine the significance of the results. (*P < 0.05, **P < 0.1).

**Figure 4:**
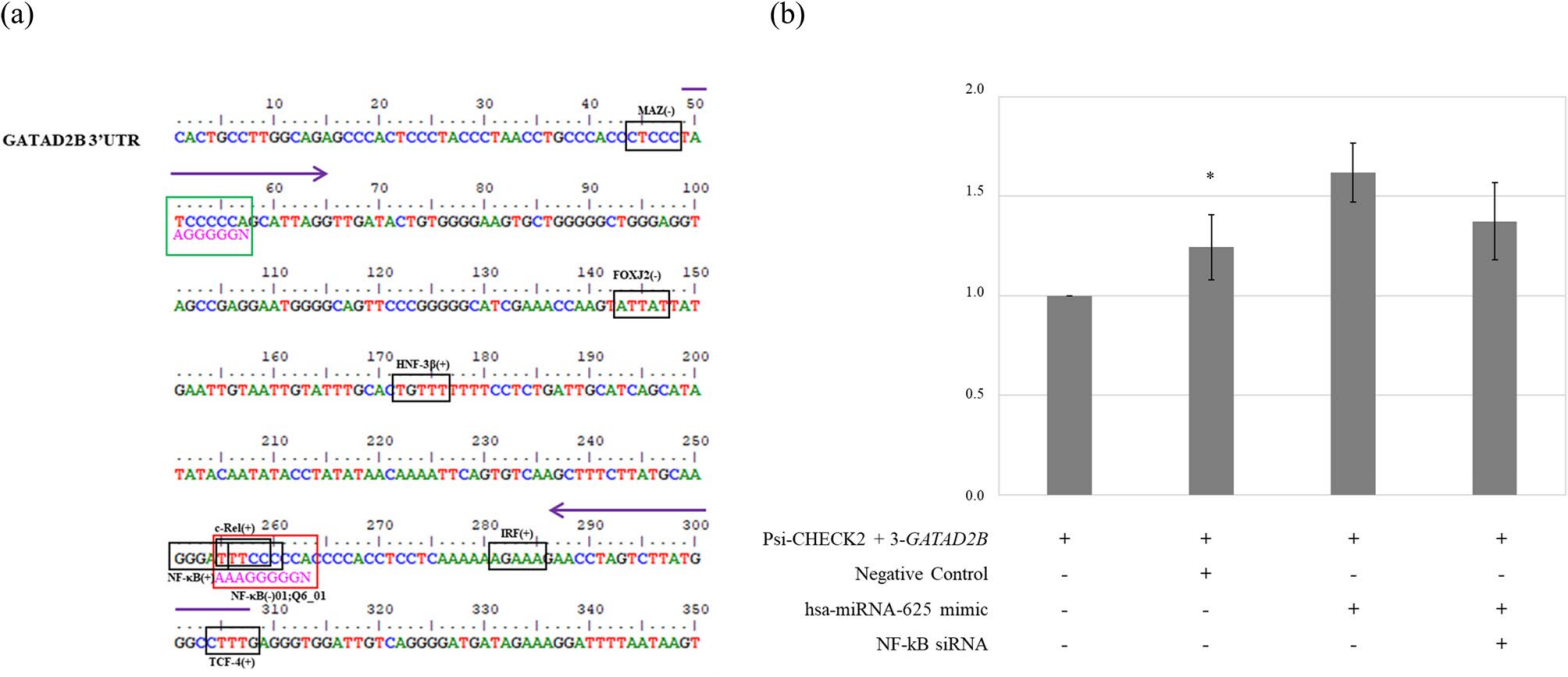
(**a**) Prediction of transcription factor binding sites near hsa-miRNA-625-5p 9mer and 7mer-A1 binding sites in the 3′UTR of *GATAD2B.* The predicted transcription factors are in black boxes with name on the top. The hsa-miRNA-625-5p 7mer-A binding site is in the green box with sequence AGGGGGN, and the 9mer binding site is in the red box with sequence AAAGGGGGN. The arrows show the location of *GATAD2B* #3 forward and reverse primers. (**b**) Luciferase analysis of *GATAD2B #3* co-transfected with hsa-miRNA-625-5p and NF-κB*.* All the bars in the graphs were plotted as the mean ± SD. The student’s *t*-test was used to determine the significance of the results. Asterisks indicate the significance of the results. (*P < 0.05).

**Figure 5:**
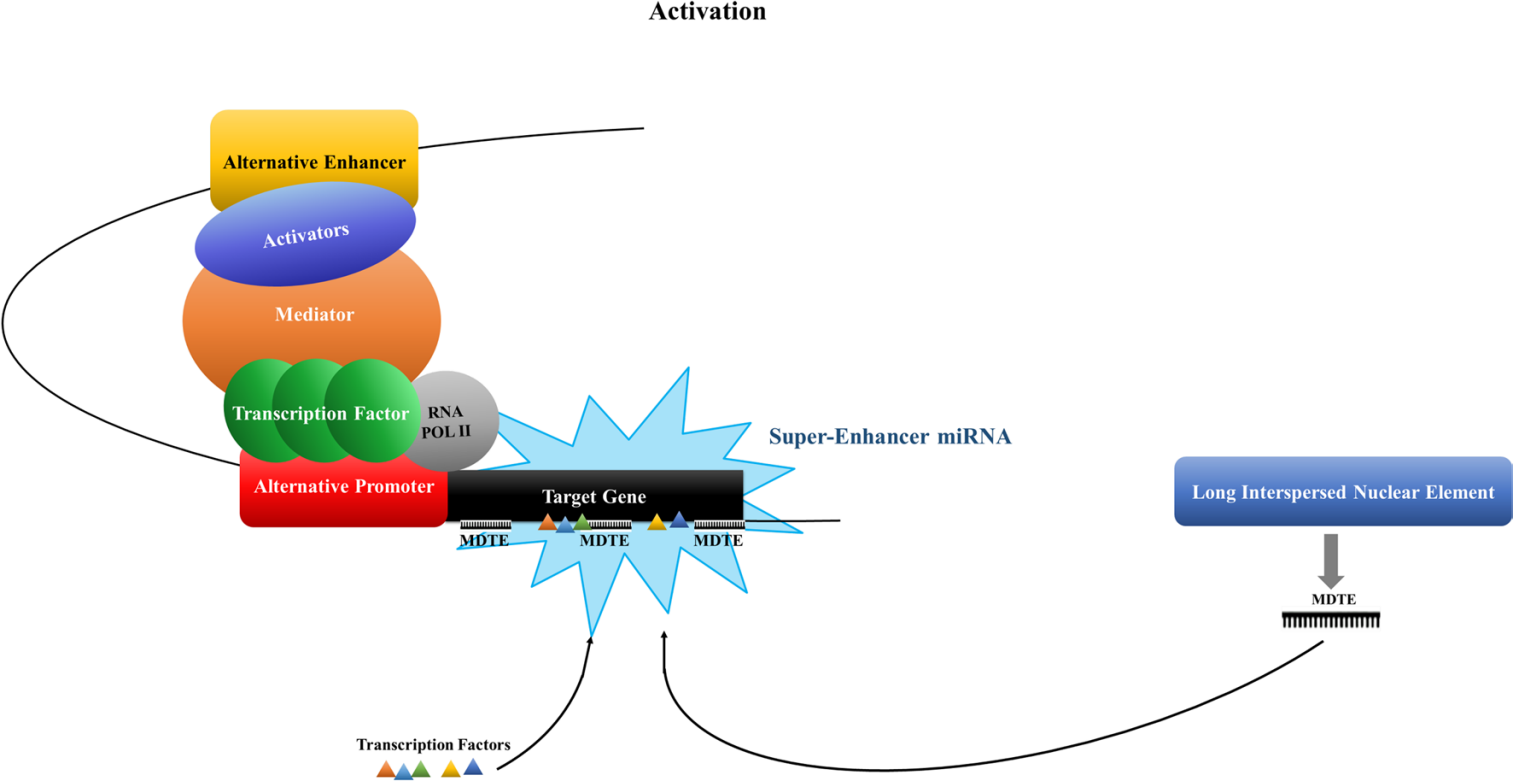
Schematic illustration of overall summarization on enhancer activity of hsa-miRNA-625-5p and NF-κB in 3′UTR of *GATAD2B*. The theory of the MDTE providing the sequence to TFs and them cooperating to grant super-enhancer miRNA ability to the MDTE.

The comparison of measured expression values from all hsa-miRNA-625-5p and four primers of *GATAD2B* showed that human samples had the highest expression in the testis of #2 primer of *GATAD2B* of 0.25, followed by the lung of #1 primer of *GATAD2B* with approximately 0.22, and then the lung of #2 primer of *GATAD2B* with 0.2. The primer #3 of *GATAD2B* showed very low expression at 0.0004. For western chimpanzee males, hsa-miRNA-625-5p showed the lowest expression at 0.0009, and similar to human samples, the *GATAD2B* #2 primer had the highest expression value of 0.25 in the cerebellum. Western chimpanzee females had the lowest expression in hsa-miRNA-625-5p and primer #3 of *GATAD2B*. The highest expression value was observed in the ovary and stomach with approximately 0.8 of primer #3, which was similar to that of humans and western chimpanzee males. The expression value for the crab-eating monkey was highest in the #1 and #2 primers of *GATAD2B*. The pancreas was the highest in #1 primer with about 0.28, followed by the spleen with over 0.25. The expression value of the mouse was highest for the *GATAD2B* #2 primer with over 0.08 in the testis. The expression level of hsa-miRNA-625-5p was low at 0.0005.

The comparison of the four primers showed that *GATAD2B* #1 and #2 primers show higher expression in primates, except that mice only show high expression in *GATAD2B* #2. In addition, the primer designed for one 9mer binding in the back showed slight expression in humans and two chimpanzees.

### Luciferase analysis of hsa-miRNA-625-5p and *GATAD2B*

To analyze the correlation between the 3′UTR of *GATAD2B* and different numbers of canonical sites and the binding location of hsa-miRNA-625-5p, lung cancer-derived derived A549 cells were used for co-transfection (Fig. 3). The results showed that *GATAD2B* #3 with one 9mer and one 7mer-A1 hsa-miRNA-625-5p binding site had the highest enhancer luciferase expression using the hsa-miRNA-625 mimic. Additionally, *GATAD2B* #3 with an hsa-miRNA-625-5p inhibitor clearly showed a decrease in luciferase expression compared with that observed using the hsa-miRNA-625 mimic. However, the results of *GATAD2B* #1 and 4 show irregular patterns. *GATAD2B* #2 showed a pattern similar to *GATAD2B* #3; however, the values were lower than those of the control.

### The correlation between NF-κB and hsa-miRNA-625-5p in the 3′UTR of *GATAD2B*

The sequence of *GATAD2B* 3′UTR was examined using TRANSFAC v.8.0 assuming that the enhancer activity of *GATAD2B* #3 with hsa-miRNA-625 was generated by TF. A total of 107 TFBSs with thresholds over 0.95 were found to exist in the 3′UTR of *GATAD2B*. TFs were labeled in the sequence of *GATAD2B* #3 with hsa-miRNA-625-5p 9mer and 7mer-A1 seed regions (Fig. 4a). Near the end of the 3′UTR of *GATAD2B*, where the 9mer hsa-miRNA-625-5p binding site is located, there are two types of TFs sharing the seed region, NF-κB and c-Rel. FoxJ2, HNF-3β, and IRF are also found in the 3′UTR of *GATAD2B* near primer #3. The result of the hsa-miRNA-625 mimic with NF-κB siRNA co-transfection showed that luciferase expression decreased compared to that observed in case of the hsa-miRNA-625 mimic without NF-κB siRNA (Fig. 4b).

## Discussion

Previous studies have suggested that MDTEs are important in evolution and diseases development, e.g., cancer; however, only a few MDTEs have been deeply analyzed and reported^14,15,39–43^. Several reports of hsa-miRNA-625 have focused on human disease and cancer, suggesting that hsa-miRNA-625 is oncomiR^24–28,44,45^. Reports dealing with miRNAs till date, have not considered the difference between the number of canonical sites in miRNA and the location of miRNA binding in the target gene when selecting target genes. However, some previous studies mentioned the importance of the quantity of canonical sites^22,46–48^. This study suggests that the number of canonical sites and the location of miRNA binding sites are important in miRNA studies because of the effect of the surrounding sequences. The 9mer of hsa-miRNA-625-5p that complementarily binds near the end of the 3′UTR of *GATAD2B* provides the binding location to NF-κB. In addition, to examine the expression pattern of oncomiR MDTE in various species at a glance, qPCR was used to analyze the relative expression of hsa-miRNA-625-5p and *GATAD2B* in humans, chimpanzees, crab-eating monkeys, and mice.

Studies on hsa-miRNA-625 have identified target genes related to human disease, such as high mobility group A1 (*HMGA1)*—which causes chromosome instability when it is deregulated—estrogen receptor 1 (*ESR1)*—which interacts with peroxisome proliferator-activated receptor gamma—coactivator 1 beta (*PPARGC1B)*—negative regulator of inflammatory signaling pathways via integrin-linked kinase (*ILK*), which is involved in signal transductions—and mutations in *ILK* as being correlated with cardiomyopathies^27,44,45^. Bioinformatic tools identified *GATAD2B* as the target gene of hsa-miRNA-625-5p as it harbors the highest number of hsa-miRNA-625-5p binding sites in its 3′UTR (Table 1). Two of each 9mer and 8mer, and three of 7mer-A1 hsa-miRNA-625-5p bind to and numerous TFs embedded in the 3′UTR of *GATAD2B.*

Primers were designed to examine the relative expression of different quantities of hsa-miRNA-625-5p seeds and binding locations in the 3′UTR of *GATAD2B* in various species, including humans, chimpanzees, monkeys, and mice. Initially, the first hypothesis was that the position of the miRNA binding site will not affect the expression; however, the quantity of seed binding numbers will affect the expression. *GATAD2B* #3 primer with one of each 9mer and 7mer-A1 was highly expressed in all species. The heatmap of relative expression data showed that hsa-miRNA-625-5p expression was not as highly expressed in all human, chimpanzees, and mouse tissues, except for the uterus in humans and slight expression in crab-eating monkey tissues (Fig. 2). Importantly, *GATAD2B* primers with one or two 8mer hsa-miRNA-625-5p binding sites in front of the 3′UTR show several highly expressed patterns, excluding *GATAD2B* #1 for mouse. From these data, we assumed that the degree of seed binding did not affect the relative expression; however, the location of miRNA binding sites in the 3′UTR affect target gene expression. The relative expression data mainly deals with the front of the *SHISA7* 3′UTR, and in the evolutionary aspect, the location and quantity of miRNA binding site matter because of low expression of *GATAD2B* #1 in mice.

Another hypothesis was that the luciferase assay of each designed primer for *GATAD2B* will reveal different activity due to dissimilar hsa-miRNA-625-5p binding seed sites and quantity of canonical sites. Most miRNA studies have shown that miRNAs inhibit the expression of target genes; however, there are studies that show enhancer activity in miRNAs with target genes^25,49^. One study on miRNA-625-3p showed that when the miRNA-625-3p mimic was treated with astrocyte elevated gene 1 (*AEG-1*), the activity was elevated^25^. There is another report that provides evidence of enhancer activity induced by miRNA, and the quantity of miRNA seed bindings and the location of miRNA binding sites influence target gene expression. The relative expression data for one of each 9mer and 7mer-A1 hsa-miRNA-625-5p binding sites showed low expression values; however, following hsa-miRNA-625 mimic treatment in that region, the activity was elevated, and after hsa-miRNA-625-5p inhibitor treatment, the activity decreased (Fig. 3). Further, the hsa-miRNA-625 mimic and hsa-miRNA-625-5p inhibitor treatment data showed activity higher than that of the hsa-miRNA-625-5p inhibitor and lower than that of the hsa-miRNA-625 mimic. In one study on MDTE, OF-miRNA-307 was also enhanced when co-transfected with the target gene *mkln1* due to OF-miRNA-307 surrounded by TF^49^.

Bioinformatic tools were used to predict TFBS in the 3′UTR of *GATAD2B.* Interestingly, not all hsa-miRNA-625-5p share the binding sites with TFs; however, the region with one of each 9mer and 7mer-A1 hsa-miRNA-625-5p binding site shares the sequence with NF-κB (Fig. 4a). NF-κB has been recognized as an enhancer or activator in previous studies, and NF-κB functions as an enhancer to hsa-miRNA-625-5p that binds to the 3′UTR of *GATAD2B*^32–34,50^. One report showed expression of oncomiR miRNA-221 and miRNA-222 induced by NF-κB and c-Jun in prostate cancer and glioblastoma cells^51^. In the case of OF-miRNA-307, several TFs bind near the OF-miRNA-307 binding site in the 3′UTR of mkln1 and one of TF SOX-5 cooperates with OF-miRNA-307 to become a super-enhancer miRNA (SE-miRNA)^49^. At last, the result of hsa-miRNA-625 mimic with siRNA of NF-κB shows that by inhibition of NF-κB in hsa-miRNA-625 mimic is lower than that in cells treated with only hsa-miRNA-625 mimic (Fig. 4b). These data suggest that NF-κB induced the enhancer activity of hsa-miRNA-625-5p in *GATAD2B*, and hsa-miRNA-625-5p gained the power of SE-miRNA.

To summarize, the MDTE provides the binding sequences to TFs and they collaborate to gain the ability of SE-miRNA for MDTE (Fig. 5)*.* Not all MDTEs provide binding sites for TFs; however, in this study, one MDTE, hsa-miRNA-625-5p, arranged binding sequences for NF-κB. Through the cooperation of hsa-miRNA-625-5p and NF-κB, hsa-miRNA-625-5p gains the ability of SE-miRNA, which activates the alternative enhancer and promoter to express the target gene. The focus was on the evidence that hsa-miRNA-625-5p is derived from LINE, and the reports on MDTEs have similar results in luciferase analysis. As mentioned earlier, both OF-miRNA-307 derived from ERV9-LTR and hsa-miRNA-625-3p derived from L1MCa showed enhancer activity. MDTEs are involved in recruiting enhancer activity-related factors and mediators to gain power for SE-miRNAs.

## Materials and methods

### Ethical statement

In this study, with the exception of the western chimpanzee-related experiments, experiments were carried out in accordance with the guidelines and regulations approved by the Pusan National University-Institutional Animal Care and Use Committee (PNU-IACUC). Experiments related to western chimpanzees were carried out in accordance with the guidelines and regulations approved by the Animal Experimentation Committees of the Kyoto University. The study was carried out in compliance with the ARRIVE guidelines.

### Bioinformatics analysis

The sequence of has-miRNA-625-5p (5′- AGGGGGAAAGUUCUAUAGUCC -3′) was downloaded from miRbase v22.1 (http://www.mirbase.org)^52^ and then the sequence was used to localized in human genome (GRCh38) and to download sequence of L1MCa by UCSC Genome Browser (http://genome.ucsc.edu)^53^. The seed region of hsa-miRNA-625-5p is 5′-GGGGGAA-3′ and it is in q arm of human chromosome 14 and hsa-miRNA-625-5p is overlapped with L1MCa. The sequences of hsa-miRNA-625-5p and L1MCa were aligned using BioEdit (http://www.mbio.ncsu.edu/BioEdit/bioedit.html)^54^ to compare the analogy. TargetScanHuman was used (http://www.targetscan.org/vert_72/)^55^ and the target gene candidates of hsa-miRNA-625-5p are listed (Table 1). The sequences of the *GATAD2B* gene and MIR3, located in the 3′UTR of the *GATAD2B* gene, were also downloaded from the UCSC Genome Browser (http://genome.ucsc.edu)^53^.

The structural interaction between hsa-miRNA-625-5p and the 3′UTR of *GATAD2B* was generated using BiBiServ RNAhybrid (https://bibiserv.cebitec.uni-bielefeld.de/rnahybrid)^56^. BiBiServ RNAhybrid was used to predict target genes and to analyze the MFE values, and the MFE values represent the score of binding affinity between miRNA and target genes. The sequences of *GATAD2B* and hsa-miRNA-625-5p were used to detect complementary binding sites of hsa-miRNA-625-5p in the 3′UTR of *GATAD2B*, and each differently designed primer was created using Primer3 v 4.1 (http://primer3.ut.ee/)^57^.

The putative TF binding sites in the 3′UTR of *GATAD2B* were predicted using MATCH in TRANSFAC v8.0 (http://www.gene-regulation.com)^58,59^. Threshold values greater than 0.95 in both the core match and matrix match were used for the prediction. The term matrix match indicates the quality of a match between a matrix and random part of the input sequences. The core match determines the quality of a match between the core sequences of a matrix.

Heatmapper was used to visualize the relative expression of hsa-miRNA-625-5p and *GATAD2B* primers. The scale bar indicates each analyzed qPCR data value for each species^60^.

### Human and animal samples

Total RNAs from 13 human and 10 mouse tissues were purchased from Clontech (Clontech, USA). Total RNA was extracted by using Hybrid-R (GeneAll, Republic of Korea) from 14 tissue samples of female crab-eating monkey, provided from the National Primate Research Center, KRIBB, following the manufacturer’s instructions. The Primate Research Institute provided the male western chimpanzee samples, and the female western chimpanzee samples were supported from the Kumamoto sanctuary, Kyoto University to Primate Research Institute in Inuyama, Japan, via GAIN. The experiments handling with western chimpanzee samples was taken at Primate Research Institute. The 11 western chimpanzee male and female tissues were used to extract total RNA using Hybrid-R (GeneAll, Republic of Korea).

The RNA samples quantitated by spectrophotometer, ND-1000 UV–Vis (NanoDrop, USA). The RNA was quantified as 500 ng, and reverse transcribed at the mRNA level using a PrimeScript RT Reagent Kit with gDNA Eraser (TaKaRa, Japan), and the miRNA levels were determined using the HB miR Multi Assay Kit system I (HeimBiotek, Republic of Korea).

### qPCR amplification in human, mouse, crab-eating monkey and western chimpanzee samples

The relative expression was checked from human, mice, crab-eating monkeys and western chimpanzee male and female by using SYBR Green Q-PCR Master Mix with High Rox (SmartGene, Republic of Korea) according to the manufacturer’s protocol in a Rotor-Gene Q system (Qiagen, Germany). The conditions of qPCR included an initialization at 95 °C for 2 min, 45 thermal cycles of 95 °C for 5 s, 55 °C for 10 s, and 72 °C for 15 s. The condition of standard melting ramp was ranged from 55 to 99 °C with a 1 °C rise at each step. The reference gene, glyceraldehyde 3-phosphate dehydrogenase (*G3PDH*) was used for normalization with *GATAD2B*. Each samples were amplified in triplicate, and the data were analyzed by the 2^−ΔCt^ method (ΔCt = Ct (*GATAD2B*)-Ct (GAPDH)). The ± standard deviation (SD) presented as the bars in the graph. The relative expression data was reanalyzed with Heatmapper (http://www.heatmapper.ca/)^60^. The relative expression data in graphs are provided in supplementary figures 1–5.

The cDNA samples for miRNA were then used for relative expression analysis by performing qPCR with HB_I Real-Time PCR Master mix kit (HeimBiotek, Republic of Korea) following the manufacturer’s protocol. The condition of qPCR includs an initialization at 95 °C for 2 min, 45 thermal cycles of 95 °C for 5 s, 55 °C for 10 s, and 72 °C for 15 s. The condition of standard melting ramp was ranged from 55 to 99 °C with a 1 °C rise at each step. The small nuclear RNA (snRNA) U6 was used as reference miRNA for normalization of relative expression analysis for miRNA-625-5p (5′-AGGGGGAAAGUUCUAUAGUCC-3′). Each samples were amplified in triplicate, and data were analyzed using the 2^−ΔCt^ method (ΔCt = Ct (hsa-miRNA-625-5p)-Ct (U6)). The ± standard deviation (SD) presented as the bars in the graph. The relative expression data was reanalyzed with Heatmapper (http://www.heatmapper.ca/)^60^.

### Genomic DNA extraction and gene cloning

The DNeasy Blood & Tissue Kit (Qiagen, Germany) was used to extract genomic DNA (gDNA) from human cell line HEK293A. gDNA was used for PCR amplification with 2 × TOP simple DyeMix (aliquot)-HOT premix (Enzynomics, Republic of Korea). The PCR mixture contained with 10 µL of contained with 2 × TOPsimple DyeMix, 7 µL of distilled water, 1 µL of gDNA template, and 1 µL of each primers (10 pmol/µL). The condition of PCR were as follow: an initialization at 95 °C for 5 min, 35 thermal cycles of 94 °C for 40 s, primer-specific annealing temperatures for 40 s, 72 °C for 40 s, and a final elongation step at 72 °C for 5 min. The products of PCR were separated on a 1.5% agarose gel and purified with Expin Gel SV (GeneAll, Republic of Korea). The purified PCR products were cloned into a psi-CHECK2 vector (Promega, USA) by LigaFast Rapid DNA Ligation System (Promega, USA). The Exprep Plasmid SV, mini (GeneAll, Republic of Korea) was used for plasmid isolation.

### Cell culture and luciferase assay

The A549 cells derived from lung cancer, were grown at 37 °C in a 5% (v/v) CO_2_ incubator in Roswell Park Memorial Institute (RPMI) medium (Gibco, USA) added with 10% (v/v) heat-inactivated fetal bovine serum (FBS) (Gibco, USA) and 1% (v/v) antibiotics-antimycotic (Gibco, USA). The cells density of 1 × 10^5^ cells/well were plated in 24-well plate. After the incubation at 37 °C in a 5% (v/v) CO_2_ incubator for 24 h, the cells were transfected with 500 ng of cloned psi-CHECK2 vector and 100 µM miRNA mimics using jetPRIME (Polyplus, France). The cells were lysed using 1 × passive buffer (Promega, USA) after 24 h of incubation. The Dual-Luciferase Reporter Assay System (Promega, USA) was used to analyze the luciferase activities. The specific information of transfected reagents are shown in Fig. 3. The negative control mimic contains with the scrambled miRNAs. The miRNA-625 mimic is a chemically synthesized double-stranded RNA oligonucleotide, and the miRNA-625-5p inhibitor is a complementary binding sequence of a target miRNA made up of a single-stranded synthetic inhibitor. The luciferase analysis performed in triplicate, and data with bars represent the mean ± SD. To determine the significance of the results, the Student’s *t*-test was performed using Excel (Microsoft Corp.) (*P < 0.05, **P < 0.1).

For the additional study, 100 µM of NF-κB siRNA (Bioneer, Republic of Korea) was used in co-transfection of the *GATAD2B* #3 plasmid vector and hsa-miRNA-625. Lane 1 transfected with only the plasmid vector of *GATAD2B* #3. Lane 2 transfected with *GATAD2B* #3 plasmid vector and co-transfected with negative control mimic. Lane 3 transfected with *GATAD2B* #3 plasmid vector and hsa-miRNA-625 mimic. Lane 4 transfected with *GATAD2B* #3 plasmid vector and hsa-miRNA-625mimic with siRNA of NF-κB. The luciferase analysis performed in triplicate, and the bars in the data represent the mean ± SD. To determine the significance of the results, the Student’s *t*-test was performed in Excel (Microsoft Corp.) (*P < 0.05).

## Supplementary Information

Supplementary Information 1.

Supplementary Information 2.
