# Supplementary material for: The enhancer activity of long interspersed nuclear element derived microRNA 625 induced by NF-κB
Source: Sci Rep. 2021 Feb 4;11:3139. doi: 10.1038/s41598-021-82735-x (PMC7862687; doi:10.1038/s41598-021-82735-x)
Supplement: Supplementary file 1 — Supplementary Information 1. [file 41598_2021_82735_MOESM1_ESM.docx]

**The enhancer activity of long interspersed nuclear element derived microRNA 625 induced by NF-κB**

Hee-Eun Lee^1,2,3^, Sang-Je Park^3^, Jae-Won Huh^3,4^, Hiroo Imai^5^, Heui-Soo Kim^2,6,^*

^1^Department of Integrated Biological Science, Pusan National University, Busan 46241, Republic of Korea

^2^Institute of Systems Biology, Pusan National University, Busan 46241, Republic of Korea

^3^National Primate Research Center, Korea Research Institute of Bioscience and Biotechnology, Cheongju 28116, Republic of Korea

^4^Department of Functional Genomics, KRIBB School of Bioscience, Korea University of Science and Technology (UST), Daejeon 34113, Republic of Korea

^5^Department of Cellular and Molecular Biology, Primate Research Institute, Kyoto University, Inuyama, Aichi 484-8506, Japan

^6^Department of Biological Sciences, College of Natural Sciences, Pusan National University, Busan 46241, Republic of Korea

^*^ Correspondence to: Prof. Heui-Soo Kim

Department of Biological Sciences

College of Natural Sciences

Pusan National University

Busan 46241

Republic of Korea

Fax: +82 51 581 2962

Tel: +82 51 510 2259

E-mail: [khs307@pusan.ac.kr](mailto:khs307@pusan.ac.kr)

**Supplementary figure**

Supplementary figure 1.

Analysis of the relative expression of hsa-miRNA-625-5p and *GATAD2B* in 13 human tissue samples. *U6* and *G3PDH* were used as the reference genes for normalization of the expression of hsa-miRNA-625-5p and *GATAD2B,* respectively.

Supplementary figure 2.

Analysis of the relative expression of hsa-miRNA-625-5p and *GATAD2B* in 11 male western chimpanzee tissue samples. *U6* and *G3PDH* were used as the reference genes for normalization of the expression of hsa-miRNA-625-5p and *GATAD2B,* respectively.

Supplementary figure 3.

Analysis of the relative expression of hsa-miRNA-625-5p and *GATAD2B* in the tissue samples of 11 female western chimpanzees. *U6* and *G3PDH* were used as the reference genes for normalization of the expression of hsa-miRNA-625-5p and *GATAD2B,* respectively.

Supplementary figure 4.

Analysis of the relative expression of hsa-miRNA-625-5p and *GATAD2B* in tissue samples of 14 female crab-eating monkeys. *U6* and *G3PDH* were used as the reference genes for normalization of the expression of hsa-miRNA-625-5p and *GATAD2B,* respectively.

Supplementary figure 5.

Analysis of the relative expression of hsa-miRNA-625-5p and *GATAD2B* in tissue samples of 10 male mice. *U6* and *G3PDH* were used as the reference genes for normalization of the expression of hsa-miRNA-625-5p and *GATAD2B,* respectively.
