# Supplementary material for: The enhancer activity of long interspersed nuclear element derived microRNA 625 induced by NF-κB
Source: Sci Rep. 2021 Feb 4;11:3139. doi: 10.1038/s41598-021-82735-x (PMC7862687; doi:10.1038/s41598-021-82735-x)

Supplementary figure 1

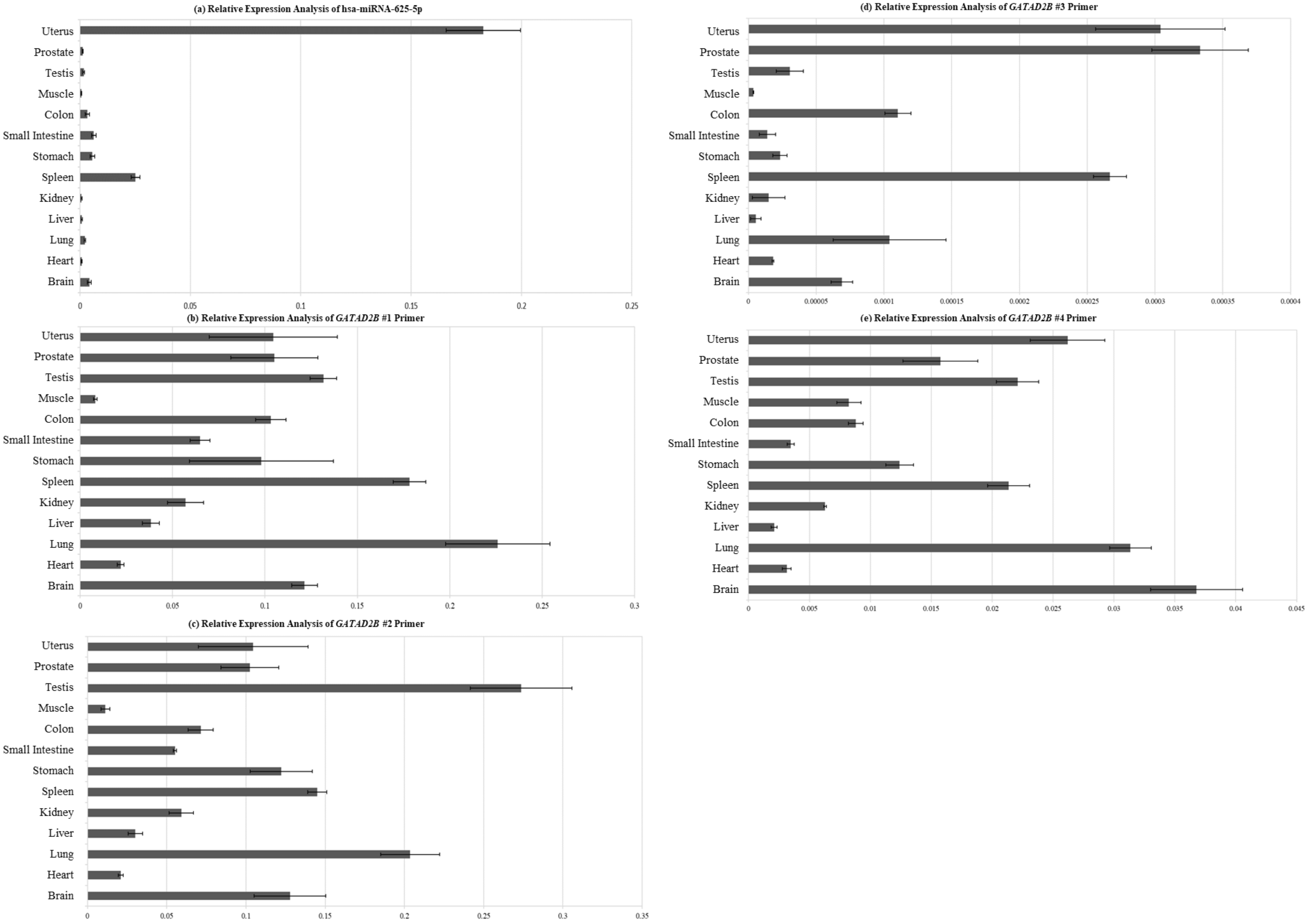

Supplementary figure 2

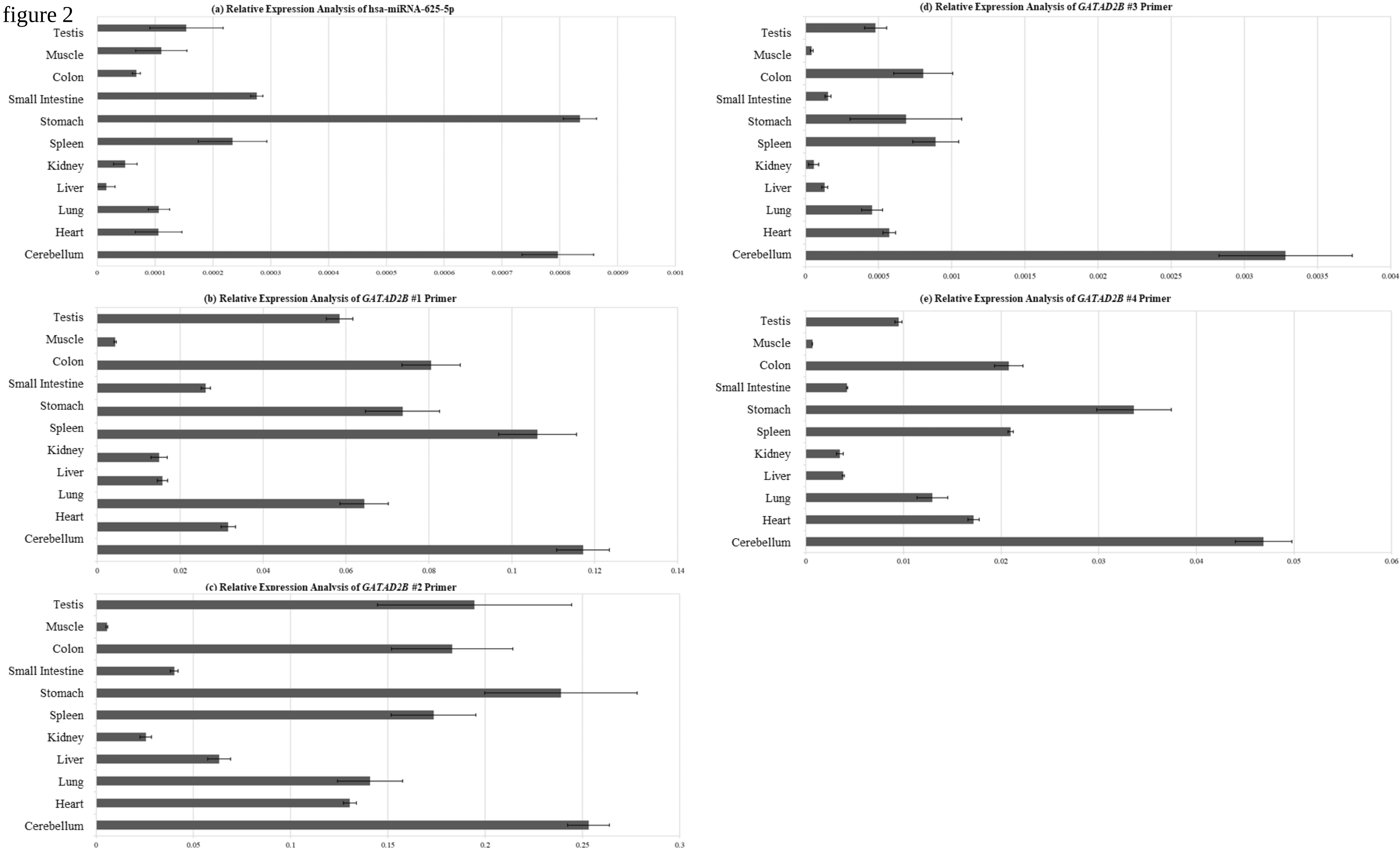

Supplementary figure 3

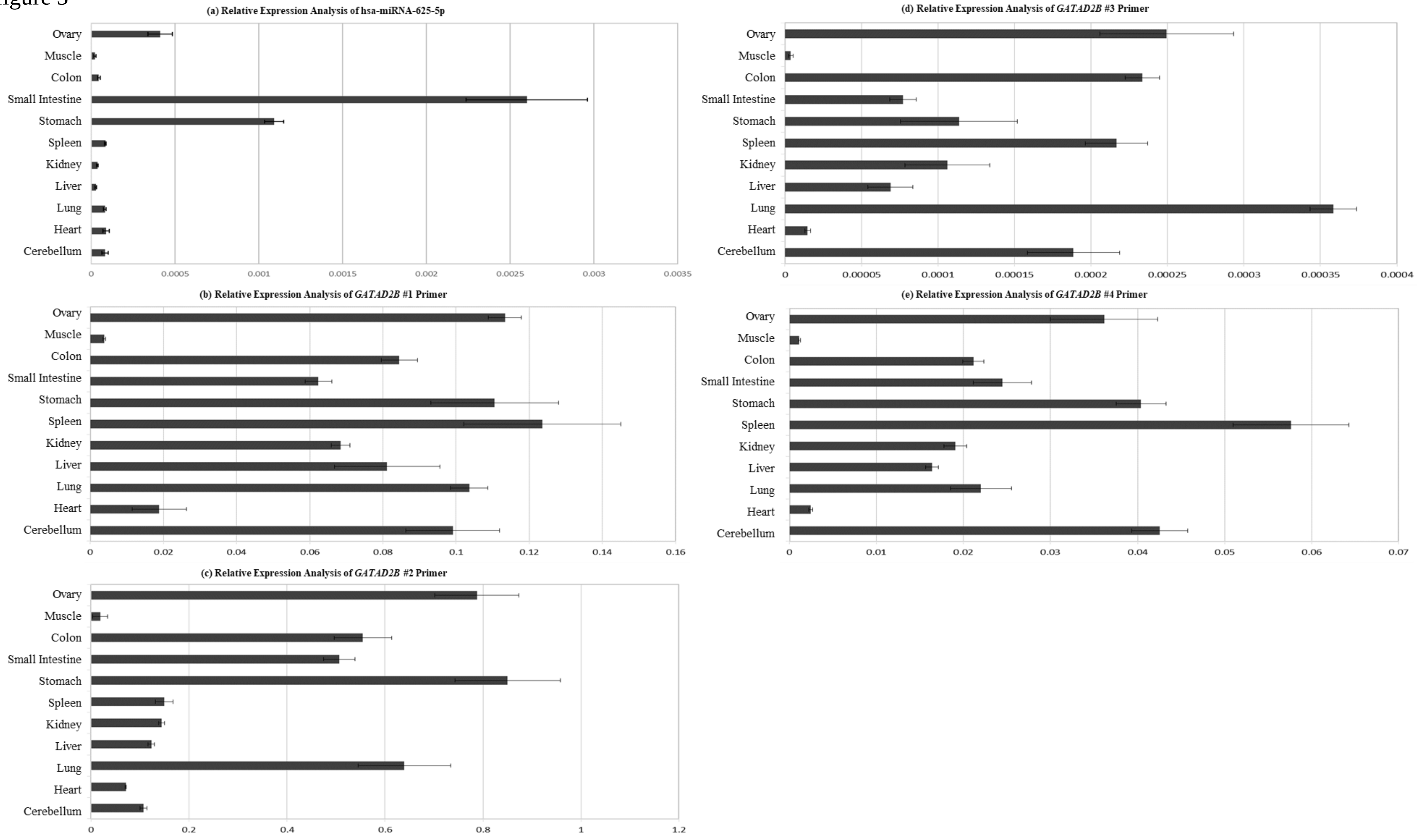

Supplementary figure 4

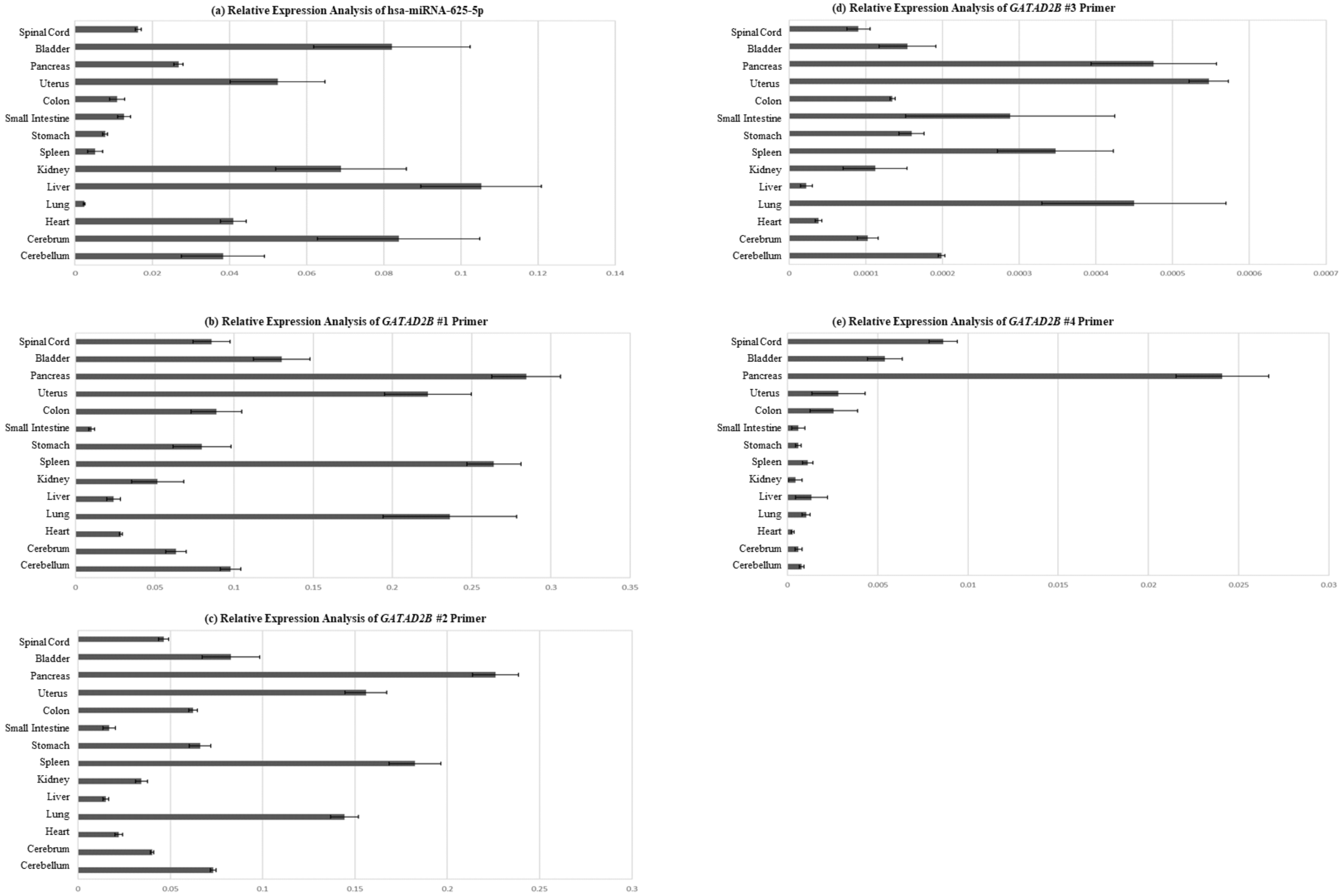

Supplementary figure 5

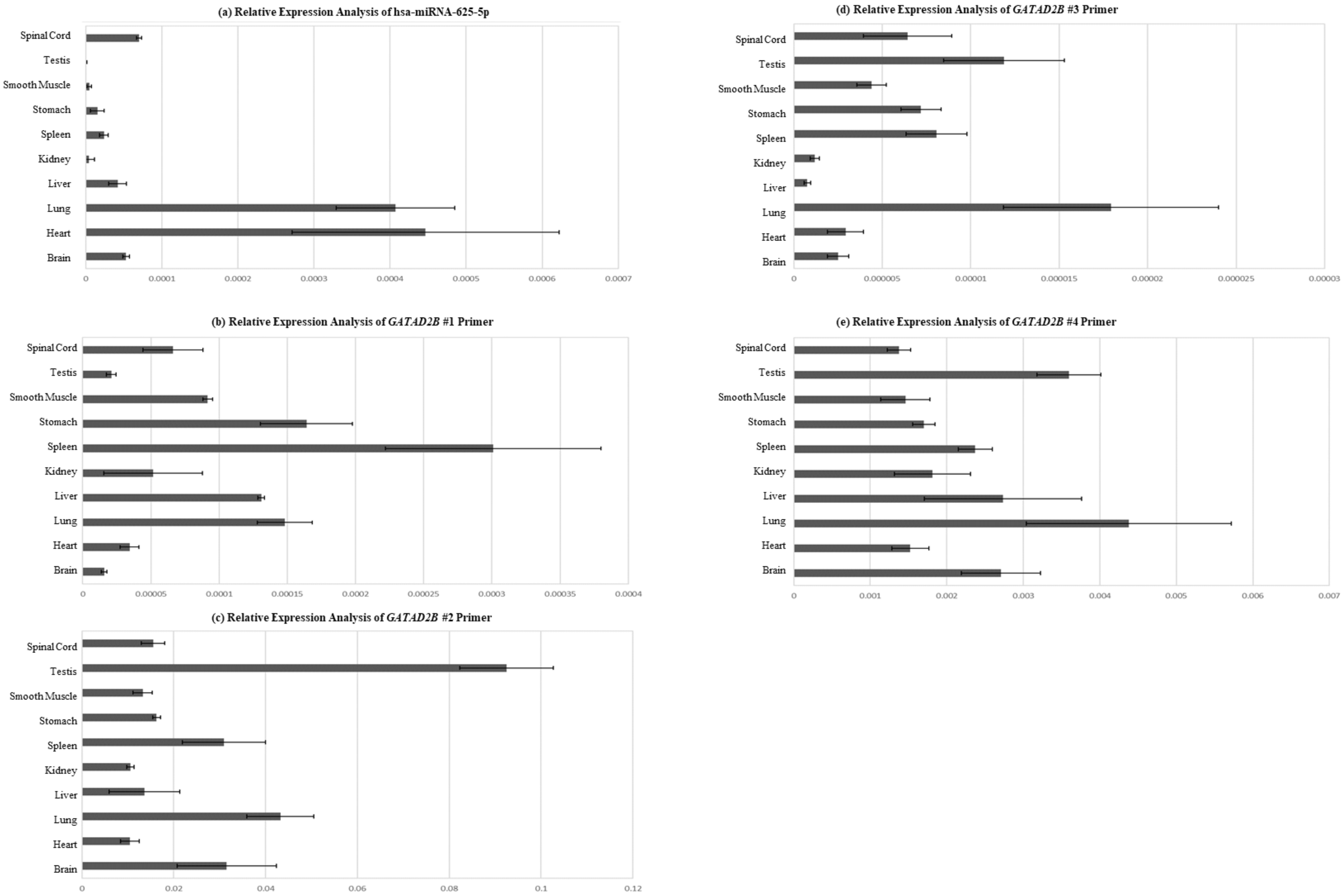

Supplement: Supplementary file 2 — Supplementary Information 2. [file 41598_2021_82735_MOESM2_ESM.pdf]
